# Extracting Multifaceted Characteristics of Patients With Chronic Disease Comorbidity: Framework Development Using Large Language Models

**DOI:** 10.2196/70096

**Published:** 2025-05-15

**Authors:** Junyan Zhang, Junchen Zhou, Liqin Zhou, Zhichao Ba

**Affiliations:** 1 Base of the State Key Laboratory of Urban Environmental Process and Digital Modelling Capital Normal University Beijing China; 2 School of Medicine and Health Management Huazhong University of Science and Technology Wuhan China; 3 Research Institute for Data Management & Innovation Nanjing University Suzhou China

**Keywords:** large language model, zero-shot prompting, information extraction, chronic disease, multimorbidity, natural language processing

## Abstract

**Background:**

Research on chronic multimorbidity has increasingly become a focal point with the aging of the population. Many studies in this area require detailed patient characteristic information. However, the current methods for extracting such information are complex, time-consuming, and prone to errors. The challenge of quickly and accurately extracting patient characteristics has become a common issue in the study of chronic disease comorbidities.

**Objective:**

Our objective was to establish a comprehensive framework for extracting demographic and disease characteristics of patients with multimorbidity. This framework leverages large language models (LLMs) to extract feature information from unstructured and semistructured electronic health records pertaining to these patients. We investigated the model’s proficiency in extracting feature information across 7 dimensions: basic information, disease details, lifestyle habits, family medical history, symptom history, medication recommendations, and dietary advice. In addition, we demonstrated the strengths and limitations of this framework.

**Methods:**

We used data sourced from a grassroots community health service center in China. We developed a multifaceted feature extraction framework tailored for patients with multimorbidity, which consists of several integral components: feasibility testing, preprocessing, the determination of feature extraction, prompt modeling based on LLMs, postprocessing, and midterm evaluation. Within this framework, 7 types of feature information were extracted as straightforward features, and three types of features were identified as intricate features. On the basis of the straightforward features, we calculated patients’ age, BMI, and 12 disease risk factors. Rigorous manual verification experiments were conducted 100 times for straightforward features and 200 times for intricate features, followed by comprehensive quantitative and qualitative assessments of the experimental outcomes.

**Results:**

The framework achieved an overall *F*_1_-score of 99.6% for the 7 straightforward feature extractions, with the highest *F*_1_-score of 100% for basic information. In addition, the framework demonstrated an overall *F*_1_-score of 94.4% for the 3 intricate feature extractions. Our analysis of the results revealed that accurate information content extraction is a substantially advantage of this framework, whereas ensuring consistency in the format of extracted information remains one of its challenges.

**Conclusions:**

The framework incorporates electronic health record information from 1225 patients with multimorbidity, covering a diverse range of 41 chronic diseases, and can seamlessly accommodate the inclusion of additional diseases. This underscores its scalability and adaptability as a method for extracting patient-specific characteristics, effectively addressing the challenges associated with information extraction in the context of multidisease research. Research and medical policy personnel can extract feature information by setting corresponding goals based on the research objectives and directly using the LLM for zero-sample target feature extraction. This approach greatly improves research efficiency and reduces labor requirements; moreover, due to the framework’s high accuracy, it can increase study reliability.

## Introduction

### Overview

Research on chronic multimorbidity has increasingly become a focal point with the aging of the population [1]. Major research directions include exploring multimorbidity patterns [2,3], investigating multimorbidity development and prediction [4,5], and examining the mutual interplay between multimorbidity and patient characteristics [6]. Amid ongoing research on frequently occurring diseases, extracting patient characteristics holds paramount importance. However, traditional machine learning–based feature extraction methods often rely heavily on extensive sample sizes for model training, and in some cases, even necessitate manual annotation. This renders the task of extracting patient features, albeit just 1 aspect of the research, not only laborious and time-consuming but also susceptible to errors [7]. To address this, we developed a framework specifically tailored for extracting features related to patients with multimorbidity. Leveraging the powerful capabilities of natural language understanding and the automation features of large language models (LLMs), this framework aims to expedite the feature extraction process, minimize extraction errors, and efficiently handle vast amounts of electronic health records (EHRs). Consequently, it significantly reduces the time and human resources required for subsequent research on comorbidities, enabling more efficient and accurate insights into this complex field.

In the task of feature extraction for patients with multimorbidity, natural language processing (NLP) technologies hold significant advantages, effectively addressing challenges such as participant scarcity and the intricacies of information extraction. Recently, LLMs have garnered significant attention across various domains, including NLP, biomedical sciences [8], and clinical practice, particularly after the emergence of ChatGPT with GPT-4. Through fine-tuning LLMs [9] and designing prompt modeling [10,11], researchers have successfully tackled diverse NLP tasks; for instance, Hu et al [10] developed prompt modeling specifically for radiology reports, enabling the extraction of pertinent disease information from these reports. In addition, Datta et al [11] designed a system based on LLMs using prompt modeling to automatically identify medical eligibility criteria. Inspired by these advancements, we propose using LLMs that have undergone medical information training and fine-tuning to extract features of patients with multimorbidity from EHRs.

We developed a comprehensive framework designed to extract pertinent feature information from patients with multimorbidity. This framework is rooted in ZuoyiGPT, a powerful architecture that builds upon the transformer model and has undergone extensive training and fine-tuning using extensive medical literature, clinical records, both online and offline patient visit data, and expert annotations. Compared to ChatGPT with GPT-4, ZuoyiGPT boasts more specialized medical information and clinical experience. Furthermore, unlike the Taiyi LLM [12], ZuoyiGPT has undergone more extensive training on clinical information and incorporates a user interaction platform, which enhances its utility and accessibility. The framework established in this study is highly adaptable and knowledge driven, ensuring its flexibility and relevance in various scenarios. Feasibility tests have confirmed the viability of ZuoyiGPT in extracting patient characteristics from EHRs. Moreover, the postprocessing module has enhanced the consistency and accuracy of the extracted feature information. To assess the framework’s performance in a zero-shot scenario, we evaluated the results of extracting both straightforward and intricate features from 300 EHRs. In a zero-shot setting, LLMs evaluated are able to extract patient characteristics solely based on the content and format requirements specified in the prompts, without relying on any additional demonstrations or training.

The study conducted patient characteristic extraction on a vast array of EHRs to ensure consistency, scalability, and interoperability. The capability to facilely extract multidimensional patient characteristics through prompt content holds significant value for analyzing a wide range of comorbidities. This framework allows for the aggregation of diverse combinations of multimorbidity, symptoms, and semistructured and unstructured mixed treatment recommendations from EHRs as objects for feature information extraction. We have delved into the strengths and weaknesses of the framework presented in the evaluation framework, identified potential future optimization suggestions, conducted a concise analysis, and provided research recommendations tailored to the demands of multidisease research. The main contributions of this study are outlined as follows:

1. A comprehensive framework based on zero-shot LLMs has been established for extracting demographic and disease-related characteristics of patients with multimorbidity, without the need for manual annotation. This framework categorizes the extraction of feature information from semistructured and unstructured EHRs into straightforward and intricate tasks.
2. The prompt modeling of the framework is adaptable to various tasks aimed at extracting different characteristics, offering flexibility and the ability to span across different disease domains. It can seamlessly be extended to accommodate extraction tasks pertaining to patient EHRs with diverse formats and structures.

### Background and Significance

An EHR contains a diverse array of patient information, including demographic statistics, disease profiles, and examination details. This comprehensive dataset serves as a cornerstone for numerous disease studies, facilitating clinical decision support and translational research endeavors; for example, Naimark et al [13] leveraged an EHR patient portal to help patients formulate their health care goals. Given the widespread embrace of EHRs [14], extracting pertinent information from these records has emerged as a pivotal focus of research [15]. Most information extraction efforts have primarily focused on 3 key subtasks: entity recognition, relation extraction, and event extraction [16-18]. By contrast, our study directly addresses the exigencies of chronic disease and multimorbidity research by concentrating on the extraction of multiple patient characteristics.

Numerous studies on multimorbidity [19-21] require the necessity of incorporating patient-specific and disease-specific features as inputs for predictive modeling, serving as the foundation for disease prediction modeling. Specifically, in the research on predicting cardiovascular disease risk in patients with type 2 diabetes [19], the initial step involves extracting patient age, gender, and disease codes as crucial inputs for the prediction model. Furthermore, when anticipating the future disease progression of patients with 0, 1, or 2 major chronic diseases [20], it becomes imperative to screen patients with 1 or 2 major chronic diseases, as well as those without any major chronic diseases, as inputs for the prediction. In addition, Lu and Uddin [21] highlight the importance of considering factors such as patient age, gender, disease codes, and smoking history in predicting chronic diseases. Consequently, the use of scientific and efficient methods for extracting disease and personal information features holds paramount importance in the study of multimorbidity.

A substantial amount of work [22-24] involves methods for extracting feature information. Kumar et al [22] used classical machine learning and deep learning approaches, using word embeddings and bag-of-words representations combined with feature selection techniques to extract features from clinical records and identify incidence rates. Kamp et al [23] adopted manual annotation to extract data from a large corpus of literature regarding the definitions, measurement methods, included conditions, and characteristics of patients investigated in studies on multiple diseases. Hu et al [24] achieved entity recognition and relationship extraction of symptoms and details within EHRs through an end-to-end overlapping joint extraction approach.

LLMs exhibit emergent behavior due to their transformer-based architecture [25]. Since the release of ChatGPT, LLMs have garnered significant attention and are currently being applied across various fields, including NLP, data analysis, medicine, and artificial intelligence. In clinical practice, LLMs can generate diagnostic lists and aid in clinical decision-making by leveraging their intelligent question-answering capabilities [26]. In the field of medical research, LLMs are able to abstract complex social determinants of health from original and unidentified medical notes [27], which can also assess the likelihood of epidemics based on the content of tweets [28]. In addition, in the realm of medical texts, these models are capable of generating patient clinical information and medical records [29]. It is anticipated that LLMs may one day facilitate real-time monitoring and predictive analysis [30].

In the medical field, numerous studies have explored the use of LLMs for NLP. A significant proportion of these studies have emphasized the fine-tuning of these models to simulate physician-patient communication, ultimately aiming to provide patients with electronic physician services [12,31,32]. Specifically, Yang et al [31] introduced the first Chinese-based medical LLaMA–based LLM, which progressed through a comprehensive process ranging from continuous pretraining (supervised fine-tuning) to human feedback reinforcement learning. Luo et al [12] focused on bilingual fine-tuning of LLMs for various biomedical NLP tasks. Furthermore, Chen et al [32] are concentrated on psychological counseling, enhancing the empathy capabilities of LLMs through immersive multiturn dialogue contexts.

In the realm of information extraction, LLMs are primarily used to extract radiology reports [10] and meta-information from scientific literature [33]. In recent research, an LLM was used to develop an eligibility criteria extraction system [11], successfully extracting eligibility criteria from clinical trial data; however, this system is designed for 9 specific diseases and extracts information from a limited number of dimensions. Current research on chronic diseases and multimorbidity is patient oriented, requiring a broader range of dimensions. Notably, there is a dearth of studies exploring the use of LLMs for extracting patient characteristic information, which constitutes the focal point of our investigation.

Although both Hu et al [10] and Datta et al [11] alluded to suboptimal extraction results arising from the use of generic LLMs, few studies have capitalized on LLMs specifically fine-tuned with medical and clinical insights, apart from those exclusively dedicated to fine-tuning or creating such models. Consequently, drawing on previous research and anticipating future prospects, this study embraced ZuoyiGPT, an LLM that has undergone extensive training and fine-tuning, incorporating literature, clinical data, and expert physician insights. ZuoyiGPT offers an interactive, web-based platform tailored for patient feature extraction. Through a feasibility testing system, we assessed ZuoyiGPT’s proficiency in extracting patient features and subsequently used it to successfully extract a comprehensive set of 84 patient features. Notably, our feature extraction framework holds the potential to significantly reduce or even eliminate the reliance on manual annotation, thereby expanding its applicability to a broader range of feature extraction tasks.

## Methods

### Data

We conducted a systematic random sampling from the EHRs of 19,000 patients with chronic diseases at the Wanping Community Health Service Center in Beijing, China, and manually extracted desensitized EHRs for 1225 (6.45%) patients. All participants were aged >40 years and had multiple chronic diseases. These EHRs included details of 41 chronic diseases, 235 symptoms, and 312 drug recommendations. Notably, 986 (80.49%) of these 1225 EHRs contained preliminary consultation information, including personal details, medical histories, family backgrounds, lifestyle habits, self-reported symptoms, drug recommendations, and nonmedication advice. Conversely, the remaining EHRs (239/1225, 19.51%) lacked initial consultation data and solely contained personal information, medical histories, family backgrounds, and lifestyle habits. We selected a combination of longitudinal and cross-sectional EHR information for our experiments. The EHRs include both semistructured and unstructured information. The semistructured information mainly comprises basic personal details of patients, such as gender and date of birth, as well as medical history; family history; and lifestyle information related to smoking, drinking, and physical activity. The unstructured information consists of 2 parts: the patient’s presenting complaints, which contain symptom information, and the physician’s diagnostic results, which include dietary and medication recommendations. Consequently, we categorized the extraction of semistructured information as straightforward feature extraction and the extraction of unstructured data as complex feature extraction.

We used two methodologies to extract straightforward features from the 1225 EHRs: (1) a rule-based algorithm integrated with symbolic NLP and (2) ZuoyiGPT. Specifically, 10 features were used for prompt design, while 5 were designated for rapid prompt calibration. Furthermore, we manually annotated straightforward extraction information from 50 (4.08%) of the 1225 EHRs—those with preliminary diagnosis records as well as those without—to evaluate the capability of the LLM to extract simple features, thereby validating the feasibility of the ZuoyiGPT. Subsequently, ZuoyiGPT was used to extract intricate features from 986 (80.49%) of the 1225 EHRs, using 10 features for prompt design and 5 for rapid prompt calibration. Of these 986 EHRs, 200 (20.3%) were manually annotated to evaluate the LLM framework’s performance in extracting patient features. Our manual annotations were completed by medical experts, and the prompt design was also guided by their expertise.

### Framework for Extracting Characteristics of Patients With Multimorbidity

#### Overview

**Figure 1:**
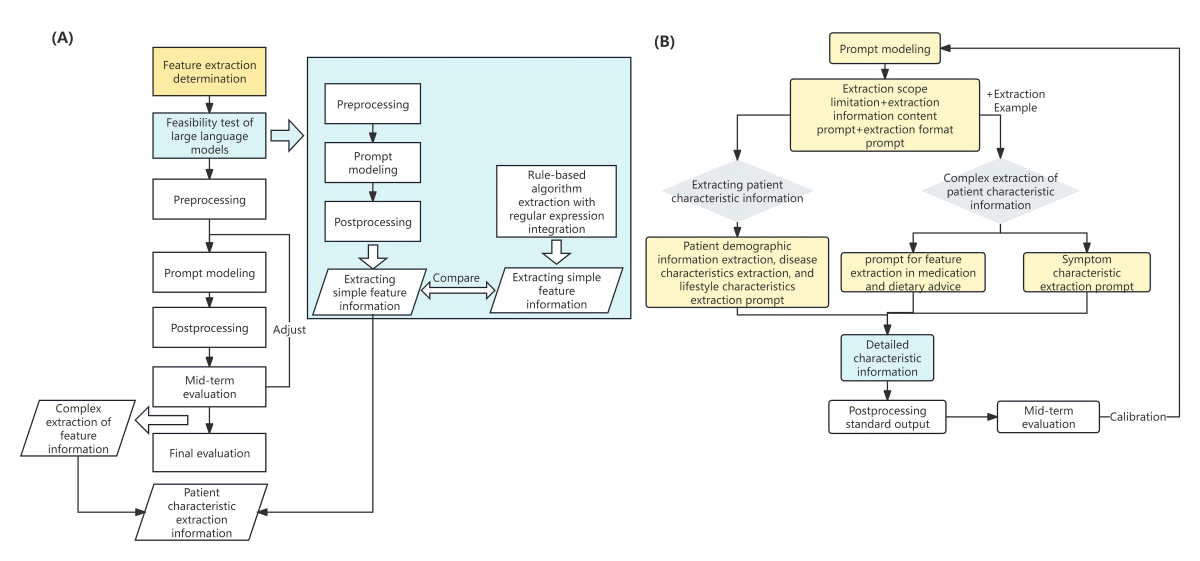
System overview.

We developed a framework for extracting multifaceted characteristics of patients with multimorbidity. Leveraging the extensive language capabilities of ZuoyiGPT, this framework automatically retrieves demographic and disease-related information from EHRs. The following subsections introduce the various components of this framework, as illustrated in Figure 1.

#### Feature Extraction Determination

**Figure 2:**
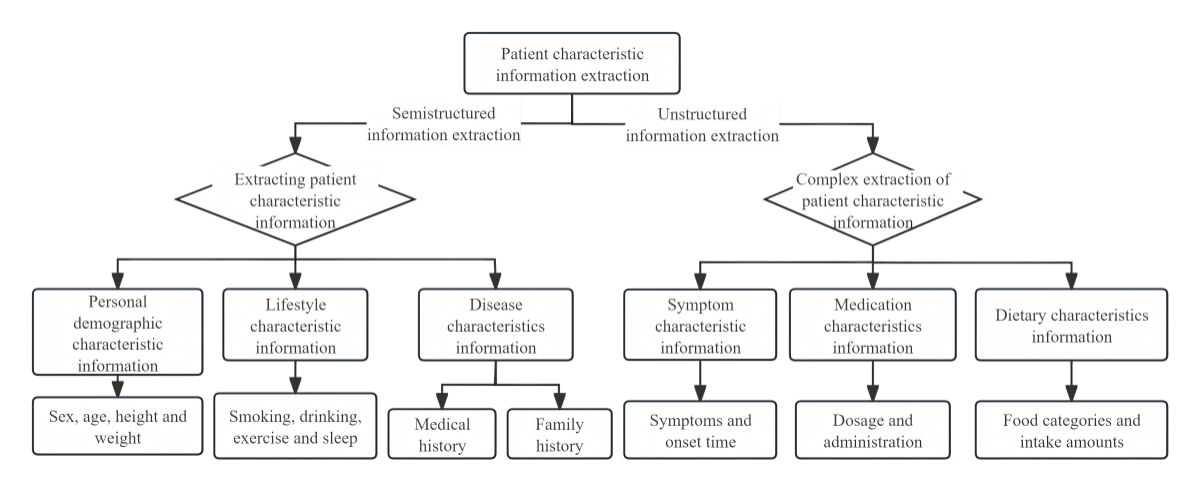
Extraction of patient characteristics.

Drawing upon insights from clinical experts and medical experts and extensive research literature on comorbidities associated with chronic and frequently occurring diseases, we arrived at a comprehensive set of extracted patient characteristic information. As outlined in the study by Cezard et al [4], research on chronic diseases typically falls into 2 categories: cross-sectional studies and longitudinal studies. Cross-sectional studies primarily focus on exploring symptoms and states as well as common risk factors, including age, unhealthy behaviors, and socioeconomic status. Conversely, longitudinal studies delve deeper into the exploration of diseases and their sequelae, tracing the trajectory of the illness over time. Dong et al [5] predicted multimorbidity through a graph convolutional network that integrated population phenotypes (including diet, physical activity, sleep, and smoking) and disease networks. Feng et al [6] investigated the effects of age and gender differences on multimorbidity. Ultimately, we opted to adopt a combined longitudinal and cross-sectional approach to studying chronic diseases. This holistic method integrates information extracted from electronic medical records, encompassing demographic characteristics, disease characteristics, lifestyle habits, symptomatic manifestations, medication profiles, and dietary patterns. This comprehensive approach aims to cater to the evolving needs of extracting patient characteristic information in future research endeavors on chronic diseases, as illustrated in Figure 2.

Demographic information comprises patient ID, gender, date of birth, age, height, weight, and BMI. Among these, patient ID, gender, date of birth, height, and weight can be directly extracted from EHRs. Nevertheless, to facilitate subsequent research endeavors, we calculated the patients’ age and BMI based on the extracted birth date, height, and weight.

Disease-related information consists of medical history and family history. The medical history includes 41 prevalent chronic diseases such as hypertension, type 2 diabetes, coronary heart disease, and stroke, along with the duration of each disease. The family history delves into 12 aspects, including whether the father or mother has coronary heart disease, diabetes, hypertension, hyperlipidemia, cerebrovascular disease, or stroke.

Lifestyle characteristic information includes smoking status, smoking duration, alcohol consumption, exercise patterns, sleep quality, and sleep disorders. Smoking status is categorized as follows: 1 signifies current smoking, 0 indicates nonsmoking, and –1 represents smoking cessation. For individuals who do not smoke or have quit smoking, smoking duration is recorded as 0. Similarly, alcohol consumption is classified as 1 for current drinking, 0 for nondrinking, and –1 for abstinence. Exercise status is differentiated into 5 categories: 3 represents daily exercise, 2 signifies exercising >3 times weekly, 1 indicates exercising 1 to 2 times weekly, 0 stands for no exercise, and –1 denotes irregular exercise. Sleep states are categorized into 3 levels: 3 for sleeping >10 hours daily, 2 for sleeping between 6 and 10 hours daily, and 1 for sleeping ≤6 hours daily. Finally, sleep disorders are dichotomized into 0 for the absence of sleep disorders and 1 for the presence of sleep disorders.

Symptom characteristic information includes a diverse range of manifestations, including dizziness, nausea, elevated blood pressure, dry mouth, headache, and 230 other symptoms. Our framework meticulously extracts the specific symptoms experienced by each patient, along with the corresponding time of symptom occurrence.

The drug characteristic information is equally extensive, encompassing enteric-coated aspirin tablets, acarbose tablets, controlled-release nifedipine tablets, atorvastatin calcium tablets, and a further 308 medications. Our framework accurately captures the medication information and dosage recommendations provided by physicians during the initial consultation for each patient. Dietary characteristic information covers 74 distinct types of food. Our framework diligently extracts the dietary categories and corresponding intake recommendations tailored by physicians during the initial consultation for each patient.

Furthermore, our framework demonstrates remarkable scalability and generalization ability by leveraging LLMs to expand its repertoire of diseases, symptoms, drug recommendations, and dietary suggestions. This enables it to continuously adapt and enhance its coverage, reflecting its sophisticated capabilities.

#### Feasibility Testing of LLMs

We used a rule-based algorithm integrated with symbolic NLP regular expressions to extract patient characteristics. These extracted results served as a benchmark for assessing the feasibility of leveraging LLMs for feature information extraction. Specifically, we used regular expressions such as “\d+\\.\d*” to identify patients’ height characteristics and “\d+\.\d*|\d{2}|\d{3}” to identify and recognize their weight characteristics, enabling us to extract pertinent demographic and disease-related information. Our algorithm initially scanned through all EHRs of patients, pinpointing and cataloging all disease descriptions mentioned. Subsequently, based on the recommendations of medical experts, we consolidated various disease descriptions; for instance, “rheumatoid arthritis” was merged with “arthropathy” and “arthritis” into “rheumatoid arthritis,” while “delusion” was merged with “mental illness” into “mental illness.” Ultimately, we identified 41 chronic diseases, some of which encompass multiple disease characteristics, as detailed in Textbox 1.

**Characteristics of chronic diseases and comorbid conditions.**

**Disease categories and descriptions of the included diseases**

- Cancer: cancer and malignant tumor
- Cerebrovascular diseases: cerebral infarction, cerebral ischemia, brain infarction, transient ischemic attack, cerebral thrombosis, cerebral arteriosclerosis, and atherosclerosis
- Mental illness: delusions and other psychiatric disorders
- Rheumatoid arthritis: osteoarthritis, arthritis, osteomyelitis
- Cardiovascular diseases (excluding coronary heart disease): heart failure, myocardial ischemia, valvular heart disease, and premature cardiac contractions
- Chronic ophthalmic diseases: bilateral retinitis pigmentosa and fundus atrophy

Subsequently, we selected 41 diseases as our benchmark and developed algorithms leveraging regular expressions to extract patient demographic characteristics, lifestyle information, disease details, and family histories from the semistructured text format.

We categorized EHRs into semistructured and unstructured information and implemented patient feature extraction and applied LLM-based extraction methods tailored to each type. Semistructured information incorporates explicit format identifiers, such as colons, carriage returns, and labels such as “personal information,” whereas unstructured information consists of patient-reported symptoms, physician-provided guidance based on patient descriptions and medical histories, as well as nonpharmacological guidance. We used the extraction of semistructured information as a feasibility test for assessing the information extraction capabilities of ZuoyiGPT. To facilitate processing by the LLM, we eliminated redundant spaces and carriage returns, simplifying the information extraction process.

On the basis of the specific information content required for extraction, we iteratively developed, tested, and calibrated a comprehensive prompt tailored as the input for ZuoyiGPT’s interactive platform. For the extraction of semistructured information, our prompt comprised 4 integral components: scope definition, extraction content prompt, extraction format prompt, and prompt query for the original text. The scope definition instructs the LLM to focus solely on extracting information from the provided text. The extraction content specification outlines the precise details that the model needs to capture. The extraction format guidance mandates the model to present the extracted information in a predefined format. The prompt query referencing the original text pertains to the segment of the EHR serving as the source material. Given the immense volume of original text involved in this information extraction task, we opted not to include an extraction example prompt. The actual prompt text and its output are illustrated in Textbox 2.

**Characteristics of chronic diseases and comorbid conditions.**

**Prompt composition**

1. Please extract the following information based on the provided content: 

2. height, weight, medical history, family history, smoking status, smoking duration, alcohol consumption, exercise habits, sleep duration, and sleep disorders.

3. Output the extracted results in the following format:

Height: XXX cm;

Weight: XX kg;

Medical History:

① Diagnosed with XXX disease in XXXX;

② ...;

Family History (1 if the parent has the respective disease, otherwise 0):

Father with coronary heart disease: 1/0

Father with diabetes: 1/0

Father with hypertension: 1/0

Father with hyperlipidemia: 1/0

Father with cerebrovascular disease: 1/0

Father with stroke: 1/0

Mother with coronary heart disease: 1/0

Mother with diabetes: 1/0

Mother with hypertension: 1/0

Mother with hyperlipidemia: 1/0

Mother with cerebrovascular disease: 1/0

Mother with stroke: 1/0

Smoking Status: Smoker/Non-smoker/Ex-smoker;

Smoking Duration (current year is 2024): XX years (0 if non-smoker);

Alcohol Consumption: Drinker/Non-drinker/Ex-drinker/Other;

Exercise Habits: Sedentary/Irregular exercise/Exercise 1-2 times per week/Exercise 3 times per week/Exercise more than 3 times per week;

Sleep Duration: XX hours;

Sleep Disorders: Present/Absent.

4. Original Text: 【Demographic Characteristics Information + Medical History + Family History + Lifestyle Habits

**Sample output**

- Height: 168.0 cm; Weight: 65.0 kg; Medical History: ① Diagnosed with coronary heart disease in August 2013; ② Diagnosed with hyperlipidemia in May 2013; Family History: Father - Coronary heart disease: Yes; Father - Diabetes: No; Father - Hypertension: No; Father - Hyperlipidemia: Yes; Father - Cerebrovascular disease: No; Father - Stroke: No; Mother - Coronary heart disease: No; Mother - Diabetes: No; Mother - Hypertension: No; Mother - Hyperlipidemia: No; Mother - Cerebrovascular disease: No; Mother - Stroke: No; Smoking Status: Non-smoker; Smoking Duration (current year is 2024): 0 years; Alcohol Consumption: Drinks alcohol; Exercise Frequency: 1-2 times per week; Sleep Duration: 6 hours; Sleep Disturbances: Present.

**Figure 3:**
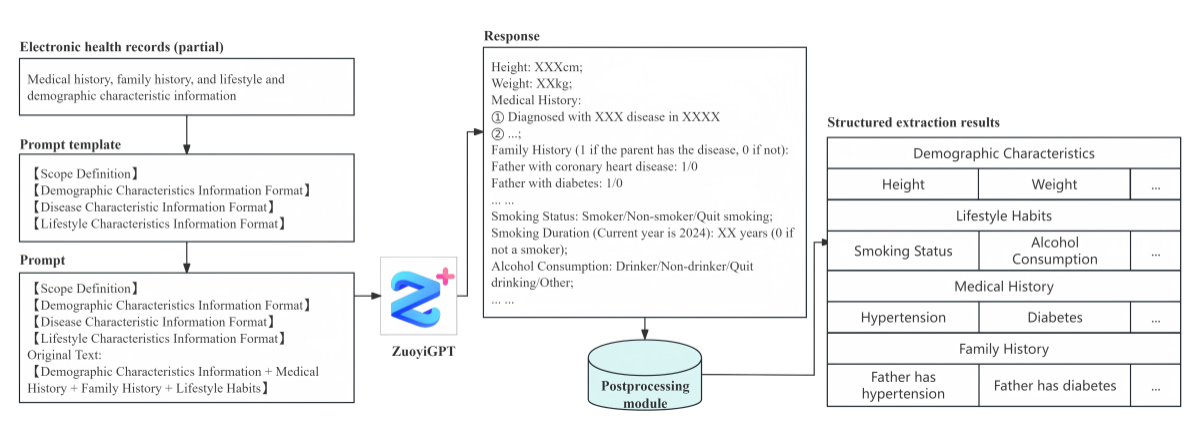
Overview of the process of simple extraction of feature information using a large language model (ZuoyiGPT).

After using ZuoyiGPT for batch information extraction from semistructured data, we resolved formatting issues in the actual extracted results using a straightforward rule-based algorithm; for instance, we corrected instances where the LLM merged 2 lines of output into a single line by adding line breaks and eliminating redundant line breaks. Subsequently, we used a rule-based algorithm to extract keywords and numerical values from the LLM’s output, along with performing numerical substitutions. As an example, we extracted “coronary heart disease” and “2013” from “coronary heart disease in 2013,” and converted “Smoking Status: Smoking” to “Smoking Status: 1.” These extracted keywords and numerical values were then assigned to their corresponding labels, effectively transforming them into a structured tabular format. Figure 3 illustrates the entire process of extracting simple patient feature information from an EHR.

Finally, from the 1225 EHRS, we randomly selected 100 (8.16%) and manually annotated their semistructured data sections, including past medical history, family history, lifestyle, and specific objective examinations. Subsequently, we conducted a comparative analysis of the extraction results obtained through a rule-based algorithm and those achieved by using the LLM on these 100 EHRs. The comprehensive final results, showing the *F*_1_-score values for both the rule-based algorithm and LLM extraction methods, are presented in Table 1.

**Table 1:** *F*_1_-score values for simple extraction of feature information using a rule-based algorithm and a large language model (LLM).

Extraction method	Basic information (%)	Lifestyle (%)	Medical history (%)	Family history (%)	Overall (%)	SD value (%)
Rule-based algorithm	98	95	99.66	99.67	98.08	1.9049
LLM	100	99	99.7	99.67	99.59	0.3656

Clearly, the results extracted by the LLM outperformed those extracted by the rule-based algorithm, with *F*_1_-score values consistently exceeding 95%, thus confirming the feasibility of using an LLM for patient feature information extraction.

#### Preprocessing

We began by considering the composition of unstructured text in EHRs and dividing each EHR into 2 sections: current illness history and medication guidance. The current illness history includes the patient’s initial self-reported information, while the medication guidance consists of 4 parts: Western medicine prescription guidance, traditional Chinese medicine prescription guidance, nondrug guidance, and physician advice. Using prompt modeling, we guided the LLM to extract pertinent symptom information from the current illness history, including symptom names and their respective occurrence times. We also extracted medication guidance and dietary advice from the medication section, including drug names, dosages, food categories, and recommended intake quantities. We used the rule-based algorithm to extract the text related to the patients’ chief complaints, as well as the sections on medication and nonmedication guidance, from the patients’ EHRs. Unnecessary formatting elements, such as extraneous whitespace and line breaks, were removed.

#### Prompt Modeling

**Figure 4:**
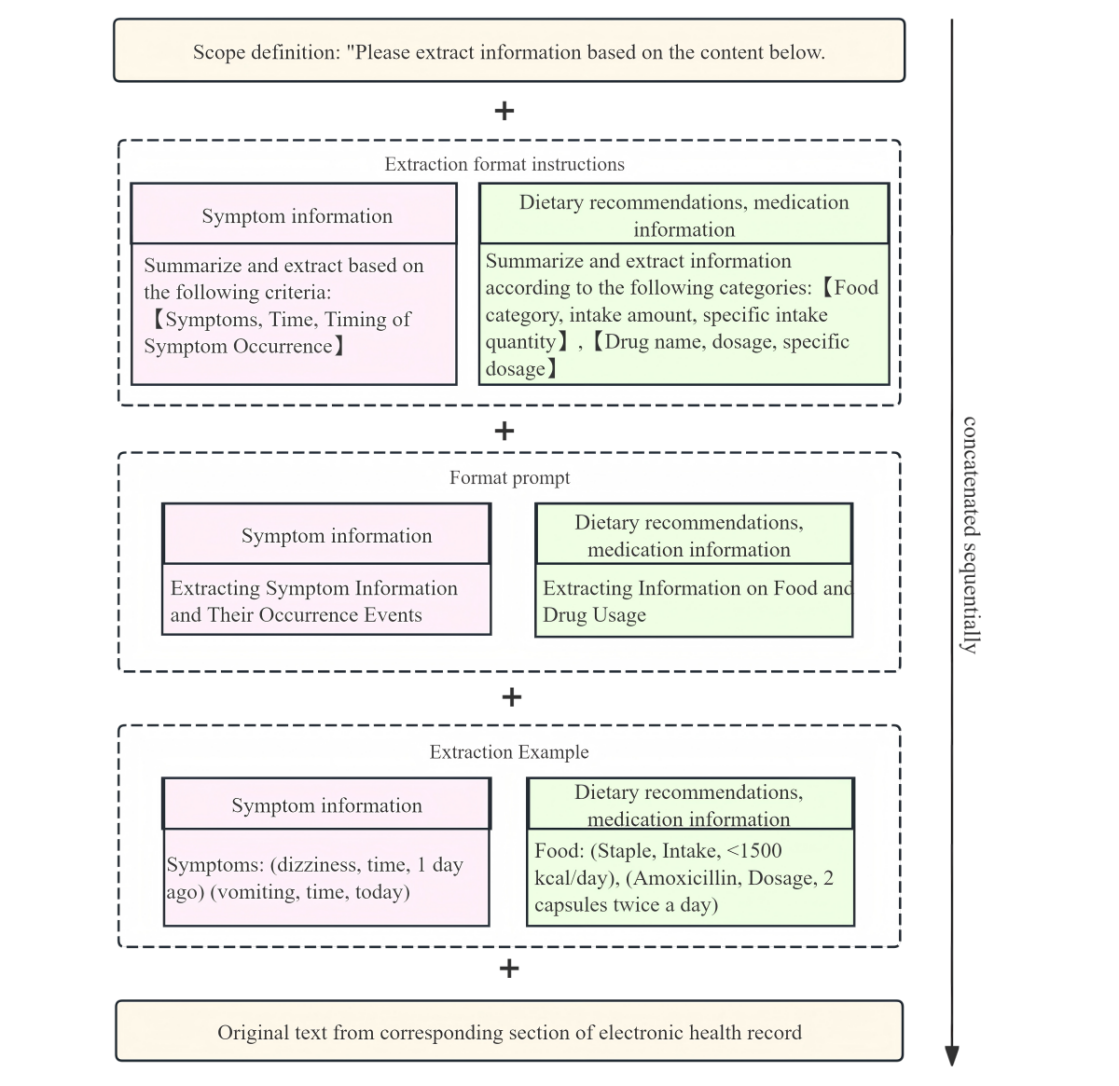
Components of complex extraction feature information prompt modeling.

Compared to complex feature extraction, the original text content for simple feature extraction is considerably more limited in scope. To guarantee accurate information extraction by an LLM, we crafted a more comprehensive prompt and fed it into the ZuoyiGPT, which excels in identifying all facets of complex feature extraction. This model underwent rigorous iterative experiments, testing, and prompt calibration. Ultimately, we developed 2 stand-alone comprehensive prompts: one dedicated to extracting symptom information and the other to extracting drug recommendations and dietary advice. Each prompt consisted of five integral components: (1) scope definition, (2) extraction format prompt, (3) extraction content prompt, (4) extraction example, and (5) prompt query text. Figure 4 illustrates the formation of the comprehensive prompt; Textbox 3 showcases example prompt templates for symptom information, along with the corresponding output; and Textbox 4 presents medication recommendation and dietary advice information, along with the corresponding output.

**Example of symptom information prompt, along with the corresponding output.**

**Prompt composition**

1. Please translate the following content.
2. Summarize and extract the food and drug information from the text in the format of: 【Food Category, Intake Amount, Specific Intake Quantity】,【Drug Name, Dosage, Specific Dosage】
3. Example: (Main Food, Intake Amount, <1500 kcal/day), (Metformin Hydrochloride Tablets, Dosage, 0.5mg / 4 times/day)

**Original Text**

【Drug Guidance + Non-Drug Guidance】

**Sample output**

【Diet】

Triples: (Low-sodium, low-fat diabetes diet, intake, no specific amount); (Sodium salt, intake, less than 6g/day); (Cooking oil, intake, less than 25g/day); (vegetables, intake, no specific amount); (Foods rich in vitamin C, intake, no specific amount)

【Medication】

Triples: (L-type amlodipine besylate tablets, dosage, 5mg once daily); (Simvastatin dispersible tablets, dosage, 20mg once daily)

**Examples of drug suggestion information and dietary advice prompts, along with the corresponding output.**

**Prompt composition**

1. Please summarize and extract the temporal information of symptoms based on the following content.

2. Organize the information in the format of 【Symptom Name, Time, Symptom Occurrence Time】.

3. For example:【Symptom: (Dizziness, Time, 2022), (Vomiting, Time, 1990)】 

4. The original text includes 【Present Illness History, Chief Complaint】

**Sample output**

- Symptoms Triplet: (Dizziness, Time, 1985) (Weakness in Lower Extremities, Time, 1990) (Palpitations, Time, 1990)
- (Sweating, Time, 1990)

Using a prompt template, we developed a web automation program that leverages an LLM to efficiently extract information from EHRs in batches and obtain their corresponding responses. For each EHR, we initiated a new conversation to prevent the extraction results from being influenced by previous dialogues. Given the potential challenge of the LLM not recognizing the output format, we opted for the ZuoyiGPT, which is trained to generate triples. Although the training format of the Left-Hand Doctor model does not exactly match our required format, it includes the format and content we need. Therefore, we decided to save the output of each EHR into separate documents and subsequently process the extraction results in a sequential manner.

#### Postprocessing

**Figure 5:**
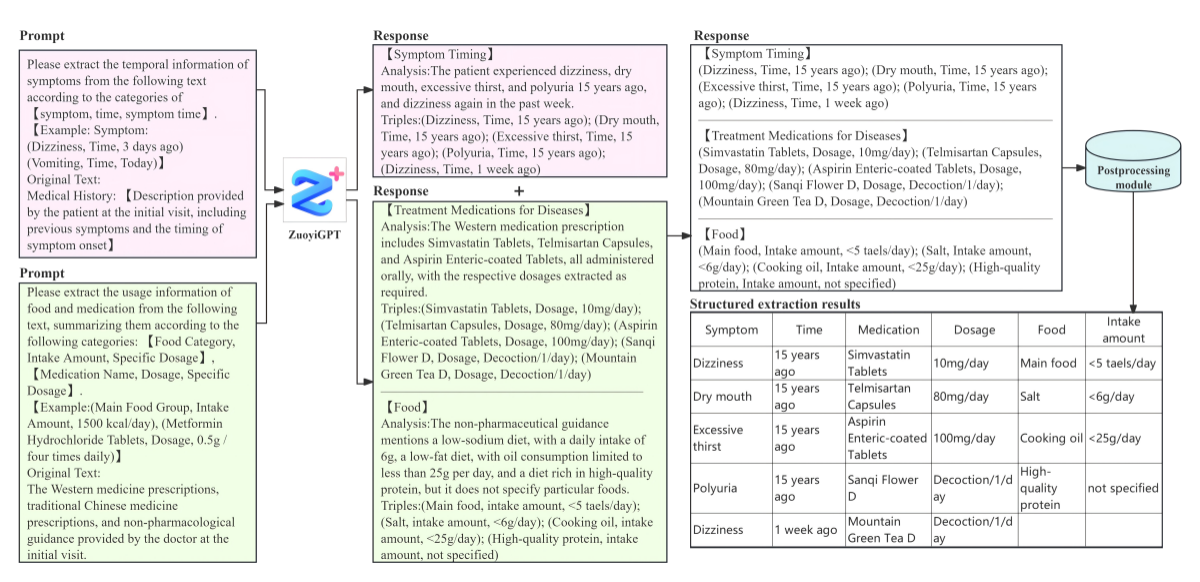
Overview of the process of extracting complex patient feature information using a large language model. The pink areas represent the extraction of symptom information, while the green areas represent the extraction of medication and dietary recommendations.

Although we provided structured output requirements, the primary purpose of LLMs is to simulate human communication. The LLMs we used have been trained in formats different from our requirements. Therefore, the results extracted by the LLM are not always in line with our expectations. To address this issue, we developed a rule-based postprocessing module with regular expressions to convert the chaotic semistructured answers into structured answers that are conducive to generating tables. Specifically, we identified the positions of all triples related to symptoms, medication characteristics, and dietary recommendations and used regular expressions to recognize the 3 parts of the triples, outputting a unified format. Each EHR involves 3 parts of triples, which are separated by specific formats and subsequently exported to individual documents for each record. Ultimately, we converted all triples into electronic medical record formats into a tabular format, ensuring consistency in the output content throughout this process; for example, terms such as “oil type,” “oil,” and “cooking oil” were unified as “cooking oil,” while “heart and brain unblocking” and “heart and brain unblocking tablets” were unified as “heart and brain unblocking tablets.” In addition, when the LLM expressed the meaning of “not mentioned,” the results could appear as “not mentioned,” “not found,” or “not mentioned.” We unified these expressions as “not mentioned” in this module and removed any “not mentioned” entries from food categories, symptom names, and medication names. Figure 5 provides a comprehensive overview of the process of extracting complex feature information from a patient’s EHRs.

### Evaluation

As part of the midterm evaluation of the patient feature extraction framework, we manually assessed the prompts and iteratively refined them based on feedback provided by medical experts. For the final framework evaluation, we conducted both quantitative and qualitative assessments. In terms of quantitative evaluation, we used 200 trial documents, manually annotated by medical experts. We reported the framework’s precision, recall, and *F*_1_-score values in identifying symptoms, symptom timing, medications, medication dosages, foods, and intake amounts. Furthermore, we calculated the framework’s accuracy in extracting complex information across 3 categories. Specifically, we reported accuracy metrics for 3 combinations: symptom+symptom duration, medication+dosage, and food category+food intake, where each label had to match precisely with manually annotated information. In addition, we initiated a new dialogue for extracting EHRs for each patient to generate more accurate responses from LLMs. The data is split into 2 sets of 100, with each set being manually annotated by a different group, and the groups then cross-validate each other’s annotations. For qualitative evaluation, we conducted a thematic analysis of missing and incorrect standard entities in 200 trials to explain the strengths and weaknesses of the patient feature extraction framework specifically tailored for patients with chronic disease.

### Ethical Considerations

This study was approved by the Ethics Committee of Tongji Medical College, Huazhong University of Science and Technology (approval number:2024 IEC (A177), as part of the project "Research on Risk Situation Awareness and Response Strategies for Chronic Disease Comorbidity Based on Knowledge Association". This study used a desensitized dataset obtained from community health service centers in Beijing, China. The collection of this dataset complied with the “ethical review measures for biomedical research involving humans”.

## Results

### Framework Performance

The framework’s performance in terms of precision, recall, and *F*_1_-score performance across symptoms, symptom duration, medications, medication dosage, food, and intake quantity is shown in Table 2.

**Table 2:** Performance measurements of 6 complex extraction features.

Features	Precision (%)	Recall (%)	*F*_1_-score (%)
Symptoms	100	97.05	98.5
Symptom duration	99.85	96.89	98.35
Medication	99.86	98.07	98.96
Dosage	77.2	94.6	84.98
Food	87.83	97.91	92.6
Intake	89.36	96.49	92.79
Overall	92.35	96.84	94.36
SD value	8.466	1.144	4.963

Table 3 demonstrates the framework’s performance in extracting intricate patient information features, particularly from unstructured textual data. Notably, the framework performed well in identifying these 6 complex feature types, with the highest accuracy for medication extraction (98.96%) and the lowest for medication dosage extraction (84.98%). The framework achieved a high overall *F*_1_-score of 94.36% across all labels. Table 3 presents the accuracy measurements for 3 combinations, revealing overall *F*_1_-score values of 98.42%, 92.64%, and 88.05%, respectively.

**Table 3:** Accuracy measurements of 3 combinations.

Feature combination	Precision (%)	Recall (%)	*F*_1_-score (%)
Symptom+symptom duration	99.92	96.97	98.42
Medication+dosage	88.99	96.6	92.64
Food category+food intake	80.38	97.35	88.05

### Extraction Accuracy

**Figure 6:**
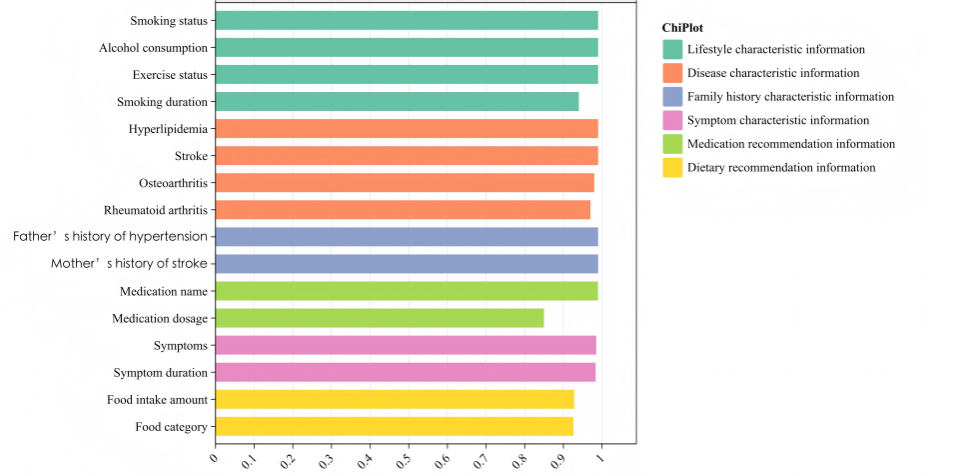
Overview of *F*_1_-score values for feature extraction.

By integrating the results of both simple and complex feature extraction, we visualized the *F*_1_-score values of all features with extraction accuracy not reaching 100%, as shown in Figure 6. Of the 67 feature labels, 61 (91%) pertained to simple extraction, while the remaining 6 (9%) concerned complex extraction. Notably, the extraction results of simple features significantly outperformed those of complex features. Of the 61 simple extraction features, 10 (16%) exhibited errors during the extraction process. These errors were observed in lifestyle-related features, including “smoking status,” “smoking duration,” “drinking status,” and “exercise status,” with *F*_1_-score values of 99%, 94%, 99%, and 99%, respectively. In the disease feature information, errors occurred in the extraction of “hyperlipidemia,” “rheumatoid arthritis,” “stroke,” and “osteoarthritis,” with *F*_1_-score values of 99%, 97%, 99%, and 98%, respectively. In addition, errors were encountered in the extraction of family history features, specifically “father with hypertension” and “mother with stroke,” both achieving an *F*_1_-score of 99%. Notably, all 6 complex features exhibited errors in their extraction.

## Discussion

### Feature Extraction Error Analysis

We developed a framework based on LLMs for extracting features of patients with multimorbidity. After completing performance measurements, we conducted an error analysis to elucidate the system’s shortcomings in each category. The *F*_1_-score values for all extracted features are visualized in Figure 6. Notably, the extraction of simple features involving unstructured text, such as “smoking status,” “drinking status,” and “exercise status,” resulted in extraction errors. Our investigation revealed that the LLM incorrectly characterized smoking status as “Smoking status: Frequent (≥5 cigarettes/day),” whereas accurate descriptions typically followed the format “Smoking status: Started in 1982, Frequent (≥5 cigarettes/day).” The absence of smoking commencement years in patient health records hindered the model’s ability to accurately infer smoking status based solely on the “smoking status: frequent” descriptor. Similarly, errors in “drinking status” stemmed from the omission of the prefix “drinking status:” in the text description, leading to misclassification and extraction by the model. The challenge with “exercise status” mirrored that of “drinking status” because it lacked the explicit textual segmentation “exercise status:”—this compromised the model’s ability to accurately extract data from partially structured texts lacking proper segmentation. When errors occurred in the semantic segmentation of the text and the semantic understanding of general knowledge, we enhanced semantic segmentation by inserting line breaks before the “smoking status,” “drinking status,” and “exercise status” during the preprocessing of medical records. A better extraction method is to use general-purpose LLMs, such as Kimi and DeepSeek, for feature extraction tasks that do not heavily involve medical background knowledge. These models are superior in their training and understanding of everyday common knowledge compared to general-purpose LLMs that are trained extensively on medical terminology at a later stage.

In disease condition recognition, LLMs exhibit a lower probability of making errors in associating disease occurrence practices with disease names; for example, in the electronic medical record of a patient where the disease is described as “2013-06, hyperlipidemia; 2008-06, stroke,” the LLM might incorrectly interpret it as indicating that the patient had hyperlipidemia in 2008 and stroke in 2013. The triggering factor for such recognition errors is unrelated to the names or categories of the diseases. Such errors can often be addressed by enhancing text preprocessing, for example, by inserting formatting separators such as a newline character or other delimiters after each disease.

In the recognition of family history, LLMs may erroneously attribute the disease status of siblings to that of the father or mother. Therefore, if a sibling has a certain disease, and this situation is described in the family history, it could lead to incorrect identification and extraction by the LLM. This issue can be addressed by incorporating the prompt “Please note the distinction between sibling illness and parental illness” into the prompt modeling or by using rule-based methods to eliminate cases of sibling illness during text preprocessing. Experiments show that adding the clarification prompt yields better results than deleting portions of the text during preprocessing.

The error rate of complex feature extraction is relatively higher than that of simple feature extraction. We categorized these errors into 6 distinct types: *incorrect extraction*, *nonrecordable*, *formatting error*, *incomplete extraction task*, *recognition error*, and *missed extraction*. *Incorrect extraction* refers to the situation where the LLM includes information that does not belong to the intended label, such as extracting “exercise regularly” into the “symptoms” label. *Nonrecordable* occurs when the LLM includes information that, while fitting the category of the label, exceeds the required range, such as extracting “clear mind” into the “symptoms” label. A *formatting error* happens when the LLM fails to output the extraction results according to the specified format, leading to missing or incorrect information in the structured table, for example, extracting “dizziness a year ago” as “(dizziness; a year ago).” An *incomplete extraction task* refers to instances where the model fails to fully extract the required content for a given label, resulting in blank entries for this label in the corresponding medical records. A *recognition error* occurs when the extraction result fits both the label category and range but does not match the actual description in the medical record, such as extracting “dizziness a year ago” as (dizziness, symptom time, a week ago). *Missed extraction* happens when the LLM completes the extraction task for the corresponding label but fails to capture all relevant information described in the medical record, for instance, only extracting (dizziness, symptom time, a year ago) from a record that also mentions “dry mouth.” The distribution of the 6 types of errors is shown in Table 4. A total of 430 (11.03%) errors were identified among the 3898 valid information entries from 200 patients, resulting in an accuracy rate of 88.97% for valid information extraction. Among the 6 types of errors, formatting errors, incomplete extraction task, and incorrect extraction were the most prevalent.

**Table 4:** Incidence of 6 types of errors in complex feature extraction.

Error types	Incorrect extraction	Nonrecordable	Formatting error	Incomplete extraction task	Recognition error	Missed extraction
Symptoms	12	10	0	0	0	0
Symptom onset	2	19	0	0	1	0
Drug	14	0	5	0	6	13
Drug dosage	18	0	119	0	4	50
Food category	9	0	23	4	10	35
Food intake	9	0	27	4	6	33

Formatting errors and incomplete extraction task are most likely to occur in drug dosages. Our error analysis revealed that the causes of these errors often related to the LLM’s poor understanding of formatting, which aligned with our previous error analysis on simple feature extraction. When outputting drug triplet information, the LLM may produce results such as (Aspirin Enteric-Coated Tablets, drug, Aspirin Enteric-Coated Tablets), as well as expressions such as (Aspirin Enteric-Coated Tablets, dosage, 100mg once daily) or (Aspirin Enteric-Coated Tablets, dosage, 100mg daily). During postprocessing, it is challenging to uniformly handle such random and varied results using rule-based algorithms. If the LLM only provides extraction results such as (Aspirin Enteric-Coated Tablets, drug, Aspirin Enteric-Coated Tablets), it becomes even more difficult during postprocessing to obtain information on drug dosages without reviewing the original electronic medical record, increasing the risk of missing or inaccurate dosage entries in the structured output table. Format output errors can be resolved using a second feature extraction method with a general-purpose language model. Specifically, after the medical LLM performs the first feature extraction, we can concatenate the formatting requirements with the first extraction results and input them into a general-purpose language model (such as Kimi, DeepSeek, ERNIE Bot, etc) to obtain feature extraction results that meet our requirements through secondary extraction from these LLMs; for example, we inputted the information presented in Textbox 5 into ZuoyiGPT.

The extraction results obtained are presented in Textbox 6.

**Example of prompt content for simple extraction of patient characteristic information, along with the corresponding output.**

- Please summarize and extract the symptom time information in the format of 【symptom name, time, symptom occurrence time】 【Example: Symptom: (dizziness, time, 2022) (vomiting, time, 1990)】. At the same time, summarize and extract food and drug information in the non-drug guidance according to the format of 【food category, intake amount, specific intake amount】, 【drug name, dosage, specific dosage】. Example: (staple food, intake amount, <1500 kcal/day), (metformin hydrochloride tablets, dosage, 0.5mg/4/day)】. Original text: Medical history: The patient experienced paroxysmal dizziness with nausea 15 years ago without obvious inducements, no vomiting, no significant activity impairment, no speech difficulties, no facial drooping, and no consciousness impairment. Blood pressure was recorded at a maximum of 160/100 mmHg, and the patient was subsequently referred to another hospital, where they were diagnosed with grade 2 hypertension. After treatment with ‘amlodipine besylate tablets, orally / 1/day, 10.0000mg each’, the blood pressure was controlled around 130-140 / 80-90 mmHg, and the dizziness symptoms significantly alleviated. Since the onset of the disease, the patient has been clear-minded, mentally stable, with normal diet and sleep, and normal bowel and bladder function.

**Medication treatment**

- Western Medicine Prescription: Clopidogrel Bisulfate Tablets, orally once daily, 1.0000 tablet per dose; Amlodipine Besylate Tablets, orally once daily, 10.0000 mg per dose; Simvastatin Tablets, orally once daily, 1.0000 tablet per dose.
- Traditional Chinese Medicine Prescription: None.
- Non-Drug Guidance: 1. Follow a low-salt, low-fat diet, controlling daily salt intake to less than 6 grams. 2. For abdominal obesity and overweight, maintain a reasonable diet, engage in moderate exercise, increase muscle mass, and enhance physical fitness. 3. Ensure a balanced diet with adequate nutrition, consume more vegetables and fruits, eat foods rich in vitamin C, reduce oil consumption to less than 25 grams per day, and limit staple food intake to less than half a kilogram per day. 4. Maintain a positive mood and reduce stress from work and life. 5. Reduce waist circumference and weight; prevent osteoporosis, falls, and accidental injuries; provide self-rescue guidance; regulate emotions, maintain psychological balance, avoid extreme emotions, maintain a balanced lifestyle, and cultivate a tranquil mind.
- Doctor’s Advice: 1. Consistently adhere to prescribed antihypertensive medication and monitor blood pressure (self-monitor weekly and keep a record; measure clinic blood pressure at least once a month), and seek medical attention for any changes in condition. 2. Regularly conduct blood tests, urine tests, stool tests, chest X-rays (anteroposterior view), electrocardiograms, liver and kidney function tests, and fundus examinations. 3. Engage in appropriate gentle outdoor activities in sunny environments, and avoid exercising in windy, cold, or snowy conditions.

**Extraction results.**

**Symptoms**

- (dizziness, time, 15 years ago)
- (nausea, time, 15 years ago)

**Food**

- (salt, intake, <6 grams/day)
- (cooking oil, intake, <25 grams/day)
- (staple food, intake, <250 grams/day)

**Medication**

- (Clopidogrel hydrogen sulfate tablets, dosage, 1/day, specific dosage, 1.0000 tablet)
- (Amlodipine besylate tablets, dosage, 1/day, specific dosage, 10.0000 mg)
- (Simvastatin tablets, dosage, 1/day, specific dosage, 1.0000 tablet)

We summarized the time information for the symptoms extracted according to the format “[symptom name, time, symptom occurrence time]”. 【Example: Symptoms: (dizziness, time, 2022) (vomiting, time, 1990)】. At the same time, we summarized the nonmedication guidance for food and medication information according to the format “[food category, intake, specific intake amount], [medication name, dosage, specific dosage]”. 【Example: (staple food, intake, <1500 kcal/day), (Metformin hydrochloride tablets, dosage, 0.5 mg/4/day)】. By concatenating the results extracted by ZuoyiGPT with the input to Kimi, a more standardized result that is conducive to postprocessing can be obtained (Textbox 7).

**Example of standardized extraction results after concatenating ZuoyiGPT outputs with Kimi input.**

- Symptom Timeline Information
- (Dizziness, time, 15 years ago)
- (Nausea, time, 15 years ago)
- Food Information
- (Salt, intake, <6 grams/day)
- (Oil, intake, <25 grams/day)
- (Staple food, intake, <250 grams/day)
- Medication Information
- (Clopidogrel bisulfate tablets, dosage, 1 tablet/day)
- (Amlodipine besylate tablets, dosage, 10 mg/day)
- (Simvastatin tablets, dosage, 1 tablet/day)

The experimental results showed that Kimi exhibited the best sensitivity to formatting.

First, the error rates and quantities for various food categories and food intake amounts exhibited remarkable similarity. Second, there were significant instances of erroneous extractions without labels; yet, these errors were distributed almost uniformly across all complex feature extractions, excluding symptom timing. We conducted separate analyses for these 2 phenomena.

Incorrect extraction of food categories can lead to erroneous extraction of food intake. Our observations revealed that, among the 4 types of feature information pertaining to drug and food information, errors in time and dosage features were as frequent as—or more frequent than—those in content features. This is because, within the same feature type, errors in content often indicate that the corresponding descriptive information is also incorrect. Therefore, if an incorrect food category is extracted, such as misidentifying smoking and drinking as food, the subsequent extraction of food intake amounts often results in extracting information about quitting smoking and drinking. Similarly, the failure to extract food categories also leads to the failure to extract food intake amounts, and the omission of food categories results in the omission of food intake amounts. LLMs have relatively chaotic definitions of “food categories” and sometimes interpret the task of extracting food recommendations as tasks unrelated to drug guidance. This has led to incorrect extraction, omission, and even incomplete extraction. However, based on input from medical experts and observations of the extraction results, we identified a consistent pattern in physicians’ dietary recommendations for patients with chronic and multiple diseases. These recommendations are typically focused on categories such as salt, cooking oil, vegetables, foods rich in vitamin C, staple foods, and foods high in dietary fiber. Moreover, the corresponding intake amounts remain almost unchanged. In other words, the description of food recommendation information is fixed, with fewer categories. Therefore, it is possible to first perform a rough extraction using an LLM and then summarize the results to provide a combination of food recommendations as a reference during prompt modeling, allowing the LLM to complete the secondary feature information extraction of food recommendations.

The issue of erroneous extraction remains consistently prevalent across 3 feature extraction categories: symptoms, medication characteristic information, and food recommendations. LLMs occasionally misclassify information into incorrect labels during the output process. Common errors include misclassifying diseases as symptoms, dietary recommendations as medication characteristic information, and lifestyle recommendations as dietary recommendations. This problem can be somewhat mitigated by incorporating error examples in the prompts.

### Comparative Experimental Study of Multiple LLMs

To further validate the ability of LLMs to extract patient features, we performed complex feature extraction experiments on data from 100 patients using 8 general-purpose or domain-specific LLMs—Kimi, DeepSeek, Tiangong, ERNIE Bot, Qwen, ChatGPT with GPT-4, ChatGLM, and Taiyi—and 1 biomedical domain model, BioBERT. For consistency and fair comparison, we applied the same prompt framework used with the ZuoyiGPT model. As shown in Table 5, the *F*_1_-score values of 8 LLMs on complex feature extraction tasks indicated that each model had its own advantage in different feature extraction scenarios. Due to extensive training on medical clinical data, ZuoyiGPT performed well and consistently in symptom and drug extraction tasks. Kimi specializes in providing extraction results that better meet user formatting requirements; however, due to inadequate training in medical data, it occasionally misdiagnosed diseases as symptoms and even produced hallucinations in drug and food extraction tasks, adding features that were not present in the text. DeepSeek showed the best overall performance, but it showed misidentification problems in the symptom recognition task and unstable performance. Tiangong and ChatGPT with GPT-4 were prone to combining multiple symptoms and tended to misclassify “aerobic exercise” as food during the food extraction task. This problem also occurred with ChatGLM when extracting food but to a greater extent: in addition to misidentifying “aerobic exercise,” ChatGLM extracted “fall prevention,” “multiple diabetes diet,” and other items as food categories. This resulted in the poor performance of ChatGLM on the food extraction task. In addition to the 8 LLMs discussed, we tested Taiyi and BioBERT. However, these 2 models lack training on targeted feature extraction and extraction format, and the extraction performance is relatively poor. Therefore, we excluded them from the results table as the performance reference for evaluating large model performance on the feature extraction task.

**Table 5:** *F*_1_-score values for complex extraction features using 8 large language models (LLMs).

LLMs	Symptoms (%)	Symptom duration (%)	Medication (%)	Dosage (%)	Food (%)	Intake (%)	Overall (%)	SD value (%)
Kimi	100	100	88.89	88.89	88.89	88.89	90.93	5.5
DeepSeek	93.61	93.61	100	100	94.74	94.74	96.12	2.58
ZuoyiGPT	98.5	98.35	98.96	84.98	92.6	92.79	94.36	4.96
Tiangong	80.6	80.6	97.44	97.44	88.89	88.89	88.98	6.88
ERNIE Bot	91.89	91.89	91.89	91.89	100	100	94.59	3.82
Qwen	90.41	90.41	100	100	92.86	92.86	94.42	4.09
ChatGPT with GPT-4	80.6	80.6	97.44	97.44	88.89	88.89	88.98	6.88
ChatGLM	96.1	96.1	100	100	46.15	46.15	97.43	29.66

**Figure 7:**
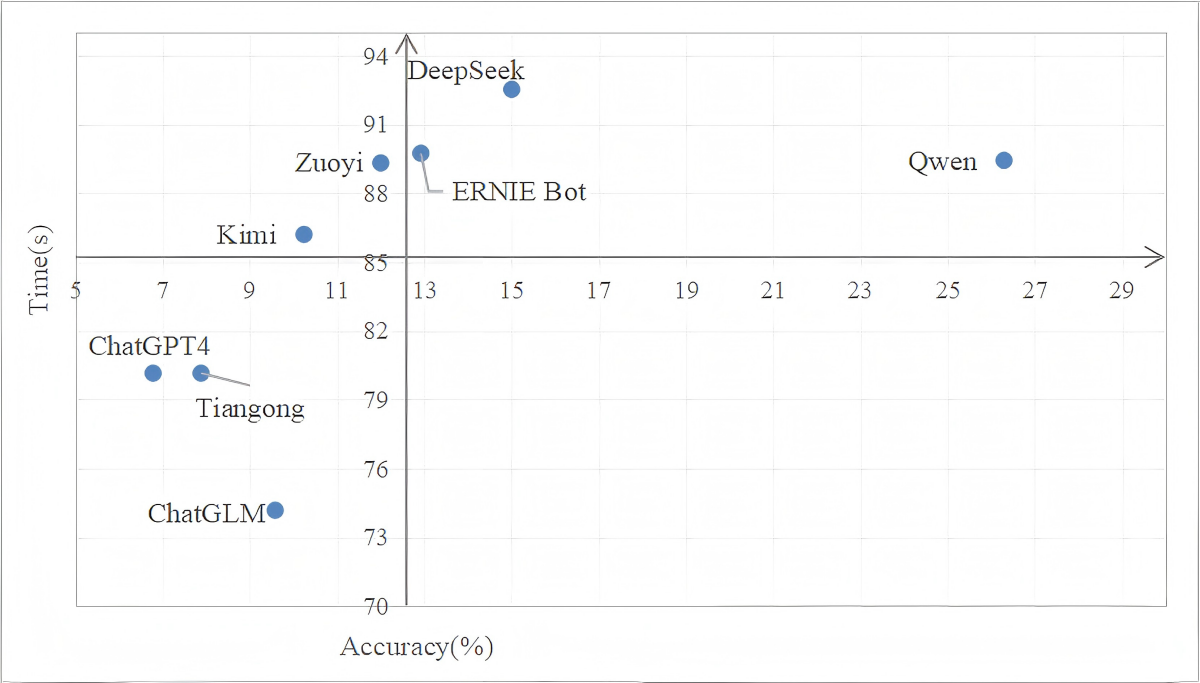
The extraction time and overall accuracy of 7 large language models.

Finally, we compiled the average time and accuracy of all complex feature extractions from the EHRs of individual patients for extracting features from 8 LLMs, as shown in Table 6. To understand their respective advantages, we integrated these tables into a chart, as illustrated in Figure 7. The performances of ZuoyiGPT and ERNIE Bot were more comprehensive, ChatGPT with GPT-4 required the least amount of time, and DeepSeek achieved the highest accuracy.

**Table 6:** The extraction time and overall accuracy of 8 large language models (LLMs).

LLMs	Time (s)	Accuracy (%)	SD value (%)
Kimi	10.24	86.2	4.32
DeepSeek	15.00	92.53	3.55
ZuoyiGPT	12.00	89.32	7.72
Tiangong	7.88	80.15	5.83
ERNIE Bot	12.92	89.74	4.23
Qwen	26.29	89.43	3.96
ChatGPT with GPT-4	6.78	80.15	7.23
ChatGLM	9.58	74.17	30.22

### A Practical Survey of Clinical and Research Applications

To validate the practicality of LLMs in extracting patient features for clinical practice, health policy, and medical research, we designed a survey and invited 2 clinical medicine experts, 2 health care policy managers, and 2 medical researchers to respond. All participants had >5 years of experience in health care–related work. The survey included questions about the application of LLMs in EHRs, the advantages and challenges of extracting patient features using these models, and the practicality of these methods in clinical practice, medical research, and the implementation and formulation of health care policies. It also solicited opinions on the accuracy of extracting patient features using LLMs and requested suggestions for method improvement. Statistical analysis indicated that, although the older medical experts and health care policy managers were not very familiar with the use of LLMs and the methods for extracting patient features, all 6 participants recognized the high practicality of these methods in clinical medicine, health care policy formulation, and medical research after using them. At the same time, all participants indicated that they would use and promote the use of LLMs to extract patient characteristic information in future work. The average κ statistic for this method was 83.41, with an average Cohen κ coefficient of 0.98 for the 2 clinical medicine experts, 0.95 for the 2 medical researchers, and 0.89 for the 2 health care policy managers. The Kendall W coefficient for the survey was 0.87, and the Cronbach α coefficient was 0.97. In summary, this method of extracting patient characteristics using LLMs is clinically valuable, and the survey demonstrated high reliability.

### Conclusions

We developed a scalable framework for extracting the characteristics of patients with multimorbidity based on LLMs capable of extracting patient characteristic information from unstructured and semistructured EHRs. This framework has the potential to be extended to new disease domains and patient populations without the need for manual data labeling, thereby facilitating large-scale analysis. In medical research, researchers can quickly extract the necessary patient characteristic information using this method without extensive training or manual involvement, streamlining the research process for multimorbidity and other disease areas, significantly enhancing research efficiency and reliability, and aiding in the construction of knowledge bases and knowledge graphs. In clinical practice, clinicians can rapidly access information about patients through this method, greatly assisting their work and treatment decisions. In addition, health care policy makers can similarly use this approach to quickly understand the characteristics of patient clusters, providing a strong foundation for the formulation and implementation of health care policies.

## Abbreviations

EHR: electronic health record
LLM: large language model
NLP: natural language processing

## Data Availability

This dataset was specifically created for this research and has not been publicly disclosed.
